# UNDERSTANDING OLDER ADULTS’ INTEREST IN USING VIRTUAL REALITY TO SUPPORT SOCIAL ENGAGEMENT ACTIVITIES

**DOI:** 10.1093/geroni/igae098.3917

**Published:** 2024-12-31

**Authors:** George Mois, Emre Eraslan, Qiyuan Cheng, Tongxin Sun, Willencia Louis-Charles, Avinash Gupta, Wendy Rogers

**Affiliations:** University of Georgia, Athens, Georgia, United States; University of Illinois Urbana-Champaign, Champaign, Illinois, United States; University of Illinois Urbana-Champaign, Champaign, Illinois, United States; University of Illinois Urbana-Champaign, Champaign, Illinois, United States; University of Illinois Urbana-Champaign, Champaign, Illinois, United States; University of Illinois Urbana-Champaign, Champaign, Illinois, United States; University of Illinois Urbana-Champaign, Champaign, Illinois, United States

## Abstract

The experience of loneliness affects one in four adults over age 65. These experiences can have significant impacts on wellbeing and quality of life. Changes associated with aging such as retirement, relocation, loss of friends and family can impact access to social engagement opportunities. Moreover, communities often lack resources required to meet individuals’ social needs. Leveraging immersive information communication technologies such as virtual reality (VR) presents a unique opportunity in bridging the resource gap, enabling individuals to connect with others, meet new people, and engage in diverse virtual activities. However, due to a limited body of research, there is little understanding of the types of VR activities older adults are interested in partaking in with in-network (e.g., family, friends) and out-of-network (e.g., strangers, new acquaintance) individuals. We explored older adults’ interests in using VR to engage with others and the types of activities they might be interested in engaging in. Participants were 21 older adults, aged 61-80 (M=69, SD=5.45). Our mixed methods approached included questionnaires, passive virtual reality demonstrations, and a semi structured interview. The older adults had positive attitudes about the VR and were interested in using it with family and friends. They could also see the potential of using VR to interact with other people with shared interests (e.g., hobbies, traveling). Participants reported interest in a diverse set of VR based activities to engage with others. These findings illustrate the potential of VR for social engagement activities for older adults, accessible from their homes, to reduce loneliness.

